# Sir Wm. Ramsay

**Published:** 1916-09

**Authors:** 


**Sir Wm. Ramsay,** the noted English chemist, who discovered argon, neon, cryton and xenon, all gaseous elements, and who transformed radium into helium, died July 23, aged 62.

